# Capacity building and predictors of success for HIV-1 drug resistance testing in the Asia-Pacific region and Africa

**DOI:** 10.7448/IAS.16.1.18580

**Published:** 2013-07-10

**Authors:** Sally Land, Julian Zhou, Philip Cunningham, Annette H Sohn, Thida Singtoroj, David Katzenstein, Marita Mann, David Sayer, Rami Kantor

**Affiliations:** 1 NRL, Melbourne, Australia; 2 The Kirby Institute, University of New South Wales, Sydney, Australia; 3 St Vincent's Hospital Sydney Limited, Sydney, Australia; 4 TREAT Asia, amfAR, The Foundation for AIDS Research, Bangkok, Thailand; 5 Department of Medicine, Division of Infectious Disease, Stanford University, Stanford, CA, USA; 6 Division of Infectious Diseases, Department of Medicine, Warren Alpert Medical School, Brown University, Providence RI, USA; 7 Conexio Genomics, Fremantle, Western Australia, Australia

**Keywords:** HIV, drug resistance, genotyping, quality assessment

## Abstract

**Background:**

The TREAT Asia Quality Assessment Scheme (TAQAS) was developed as a quality assessment programme through expert education and training, for laboratories in the Asia-Pacific and Africa that perform HIV drug-resistance (HIVDR) genotyping. We evaluated the programme performance and factors associated with high-quality HIVDR genotyping.

**Methods:**

Laboratories used their standard protocols to test panels of human immunodeficiency virus (HIV)-positive plasma samples or electropherograms. Protocols were documented and performance was evaluated according to a newly developed scoring system, *agreement with* panel-specific *consensus sequence, and* detection of drug-resistance mutations (DRMs) and mixtures of wild-type and resistant virus (mixtures). High-quality performance was defined as detection of ≥95% DRMs.

**Results:**

Over 4.5 years, 23 participating laboratories in 13 countries tested 45 samples (30 HIV-1 subtype B; 15 non-B subtypes) in nine panels. Median detection of DRMs was 88–98% in plasma panels and 90–97% in electropherogram panels. Laboratories were supported to amend and improve their test outcomes as appropriate. Three laboratories that detected <80% DRMs in early panels demonstrated subsequent improvement. Sample complexity factors – number of DRMs (*p*<0.001) and number of DRMs as mixtures (*p*<0.001); and laboratory performance factors – detection of mixtures (*p*<0.001) and agreement with consensus sequence (*p*<0.001), were associated with high performance; sample format (plasma or electropherogram), subtype and genotyping protocol were not.

**Conclusion:**

High-quality HIVDR genotyping was achieved in the TAQAS collaborative laboratory network. Sample complexity and detection of mixtures were associated with performance quality. Laboratories conducting HIVDR genotyping are encouraged to participate in quality assessment programmes.

## Introduction

Over the past decade, combined international efforts have achieved a more than 20-fold increase in access to antiretroviral (ARV) treatment for individuals infected with the human immunodeficiency virus (HIV) in resource-limited settings (RLS) [1]. Increased treatment access has been paralleled by an increased need for HIV drug-resistance (HIVDR) testing to monitor its emergence and transmission, a major threat to treatment success [2].

Whether using commercial or in-house HIVDR testing, laboratory participation in external quality assessment (EQA) is recommended by expert committees [3–7]. Available HIVDR EQA programmes include a centralized laboratory certification approach [4], a “collective” approach, with no personal communication between evaluator and evaluatees [8, 9] and “within network” and “within country” approaches, where providers liaise with participants to improve test outcome [10–14].

TREAT Asia (Therapeutics Research, Education, and AIDS Training in Asia [15]), a programme of amfAR – The Foundation for AIDS Research, is a network of more than 60 clinics, hospitals and research institutions in 13 countries working with civil society to ensure safe and effective delivery of ARVs in Asia and the Pacific [16, 17]. It also seeks to strengthen HIV/AIDS care, prevention, treatment and management skills among healthcare professionals through education and training programmes developed by experts in the region.

In 2006, TREAT Asia was funded by the Dutch Ministry of Foreign Affairs to build surveillance and monitoring capacity for HIVDR in Asia. The resulting TREAT Asia Studies to Evaluate Resistance (TASER) [18] are part of a collaborative effort: Linking African and Asian Societies for an Enhanced Response (LAASER) to HIV/AIDS, in partnership with the PharmAccess Foundation, International Civil Society Support and the AIDS Fonds [19]. While TASER is conducted in Asia, PharmAccess’ parallel Studies to Evaluate Resistance (PASER) conduct HIVDR studies in Africa [19]. With the rationale to support good quality and reliable HIVDR testing, TAQAS (TREAT Asia Quality Assessment Scheme), an EQA scheme for HIVDR genotyping, was established to support LAASER-related activities in the two regions [20]. TAQAS offered EQA of HIVDR genotyping with quantitative assessment of laboratory performance, educational feedback, follow-up of suboptimal results, trouble-shooting support and establishment of a laboratory network.

We previously described findings from the initial implementation of TAQAS within 10 laboratories over a 19-month period in 2005–2007, and demonstrated improvement or maintenance of high standards of genotyping outcomes [20]. The objectives of this study are to evaluate TAQAS performance following its expansion to include more laboratories, while testing additional EQA panels with diverse HIV-1 subtypes; develop a novel scoring system of test performance; and investigate predictors of HIVDR genotyping proficiency. A wide spectrum of laboratory expertise was included with the aim not only to evaluate, but also to improve the full spectrum of HIVDR genotyping performance, while creating a collaborative laboratory network and providing mentorship and support to emerging laboratories in RLS.

## Materials and methods

### Participating laboratories

**Figure 1:**
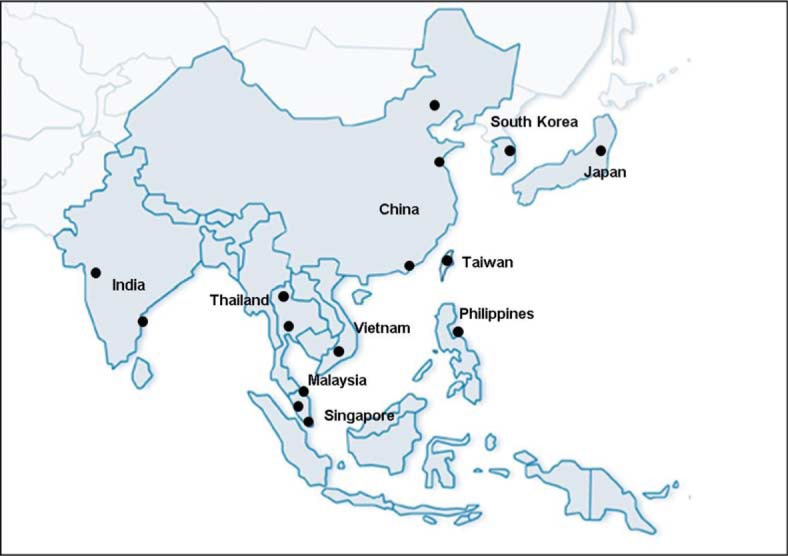
The location of TAQAS participants: China: Beijing, Shanghai, Hong Kong; India: Chennai, Pune; Japan: Tokyo (*n=*2) Nagoya; Malaysia: Kota Bharu, Kuala Lumpur; Philippines: Manila; Singapore: Singapore; Taiwan: Taipei; South Korea: Seoul; Thailand: Bangkok (*n=*3), Chiang Mai; Vietnam: Ho Chi Minh; Africa (not included in the map): Entebbe (*n=*2), Johannesburg; USA (not included in map): reference laboratory.

TAQAS participants included laboratories that provided HIVDR genotyping for clinical TASER (*n*=19 in 11 Asian countries) and PASER (*n*=3 in 2 African countries) sites (Figure 1). A Virology Quality Assessment Program (VQA)-certified US laboratory with HIVDR genotyping expertise (Stanford University) participated in an “expert” capacity as a “positive control.”

### Sample panels and testing

Over the 4.5-year study period (12/5–6/10), nine 5-sample TAQAS panels (45 samples) were distributed to and tested by participating laboratories, using their standard protocols. Panels were prepared by, shipped from and results returned to the NRL (Melbourne, Australia), an independent quality assurance provider.

Samples in seven panels were either: (i) plasma sourced from HIV-1-patients (27/35), or (ii) culture-amplified virus in HIV-negative plasma (8/35) (Table 1). Most samples contained multiple drug-resistance mutations (DRMs) and most were Subtype B sourced in Australia. Two panels (IV and VII) were *pol* electropherograms, derived from ARV-treated individuals, and used to account for inter-laboratory sequence production variation in HIVDR genotyping outcome. Panels varied in the number of DRMs; number of mixtures, defined as >1 nucleotide base at one position; and number of DRMs present as mixtures (Table 1). All plasma/virus samples (*n*=35) had a viral load >1000 copies/mL; most (31/35) >10,000 copies/mL.

**Table 1:** TAQAS panels^a^ tested and detection of drug-resistance mutations by participants between December 2005 and June 2010

			DRMs^c^ in panel samples			DRMs positions in *pol* region in panel TG			
					
TAQAS ID	Subtype(s) in panel^b^	Mixtures in panel TG	Total	Mutant	Mixtures (%)	PI	NRTI	NNRTI	Median % DRM detected (range)
I	B	73	46	35	11 (24)	V32M 46I I47V I54VM G73S V82A I84V L90M	M41L D67N L74V F116Y Q151M M184V L210W T215Y	K103N V106A	91 (80–96)
II	B and D	48	59	48	11 (19)	M46I I47V I54LMV G73S V82SI84V L90M	M41L D67N T69ins L74V M184V L210W T215Y K219N	K103H V106A V108I Y181C G190A	98 (85–100)
III	B and C	109	42	23	19 (45)	V32IM46I G48V I50V I54VT V82A I84V L90M	M41L D67N L74I V75T M184V L210W T215Y K219E	K103N V108I Y181C G190A M230L	88 (55–97)
IV (e)	A, B and CRF01_AE	46	36	29	7 (19)	M46I G48V I50V I54T V82A L90M	M41L A62V D67N K70R V75I F77L F116Y Q151M M184V L210W T215FY K219Q	K101EH K103N V106M V108I Y181C G190A	97 (89–100)
V	B and CRF07_BC	48	53	50	3 (6)	M46I I54V V82A I84V L90M	M41L D67N V75M M184V L210W T215Y	K103N	98 (96–100)
VI	B	68	59	48	11 (18)	M46LI I50L V82A	M41L D67N K70R L74V M184V L210W T215Y	K103N Y181C P225H	97 (88–98)
VII (e)	B	113	83	63	20 (24)	V32I M46I I47V G48V I50V I54T V82A L90LM	M41L A62AV D67N T69ins L74V V75T M184V L210W T215Y K219E	K101Q K103H V106A V108I Y181C G190A H221Y	90 (81–99)
VIII	B	172	38	27	11 (30)	V32I M46I I54V V82A L90M	M41L D67N L74I V75T Y115F M184V L210W T215Y K219E	L100I K103N V106I Y181C Y188L G190A	89 (76–100)
IX	B and CRF01_AE	119	56	47	9 (16)	M46I I47V I54M L76V I84V L90M	M41L K65R D67N L74V V75L M184V L210W T215FY K219E	K101EP K103NH V108I Y181C G190A H221Y	98 (91–100)

a Each panel consisted of five samples

b Non-B subtype plasma samples included one Subtype A and one Subtype C from Uganda, one CRF07_BC from Taiwan and three CRF01_AE from Thailand. Non-B electropherogram samples included two Subtype A from Kenya and one CRF01_AE from Thailand

c listed as mutations conferring HIV drug resistance in IAS-USA list and Stanford Database.

TG, target genotype; Mixtures: nucleotide mixtures; DRMs, drug-resistance mutations; PI, protease inhibitor; NRTI, nucleoside reverse transcriptase inhibitor; NNRTI, non-NRTI; e, electropherogram sample format used in panel.

Samples were shipped biannually frozen on dry ice (plasma panels) or sent electronically (electropherogram panels) to TAQAS participants after obtaining necessary country-specific permits. Within five weeks of panel receipt, participants were required to return three result outputs: (i) FASTA nucleotide *pol* sequences; (ii) lists of DRMs; and (iii) predicted susceptibility to a standardized list of ARVs. Detailed genotyping information on testing methods was collected via an electronic “Protocol Questionnaire” (Supplementary File 1).

### Laboratory performance evaluation

Sequences returned underwent quality control and phylogenetic analyses to examine within-sample clustering using Sequence Quality Analysis Tool (SQUAT) [21]. Outlier sequences (bootstraps <99%) that did not cluster appropriately were manually inspected for mutation motifs and mis-alignment, and if consistent, they were reported and omitted from further analyses.

HIVDR genotyping performance was evaluated against a panel-specific consensus sequence, termed the “target genotype” (TG), deduced by aligning sample-specific FASTA nucleotide sequences returned by all participants and applying an algorithm to identify the most likely consensus quasi-species detectable by DNA sequencing [22]. The algorithm considered that it was extremely unlikely for multiple participants to incorrectly sequence the same nucleotide at any single position; therefore, nucleotide mixtures reported by two or more participants were included in the TG. DRMs were defined as mutations listed in both the Stanford Resistance Database [23] and the International AIDS Society-USA list [24]. When participants were unable to return a complete set of panel results due to amplification or quality issues, analyses were executed only for completed samples that satisfied the quality control analyses.

Laboratory performance was scored using a system composed of eight criteria (Table 2). Performance was not evaluated by the scoring system if participants reported results from fewer than four out of five samples per panel because of failure to amplify sequences, and/or production of poor quality sequences as deemed by the participant and/or the quality control analyses indicative of potential sample mix-up or contamination. “No Score” was by definition a poor performance outcome.

**Table 2:** Scoring system developed and applied to evaluate participants’ HIVDR genotyping outcome

Criteria name	Criteria detail	Penalty points		Evaluation context
(i) Participation	Submission of any results	10 points if fail to submit any result One point per week of late submission	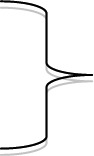	Suitability of testing turnaround time for patient management and/ or research purposes
(ii) Nucleotide sequence submission^H^	Submission of both PR and RT sequences	One point for each failure to submit PR or RT sequences per sample; *NO SCORE* if sequences from <4 of 5 samples returned	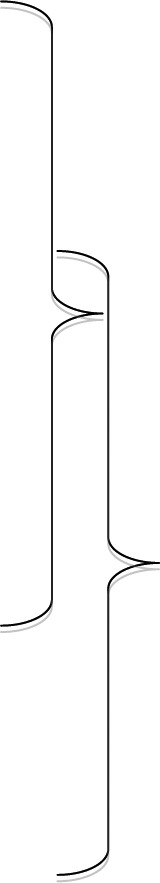	
(iii) Sequence clustering^H^	Adequate sequence clustering with same-samples from other participants	Five points per PR or RT inadequate clustering; *NO SCORE* if sequences from >4 of 5 samples do not cluster		Disqualified from analysis if insufficient or outlier sequence data are returned
(iv) Nucleotide sequence agreement	Level of agreement (%) with the complete target genotype (TG) (consensus sequence)	≥99% agreement: 0 points 98 to<99% agreement: 2 points 95 to<98% agreement: 5 points 90 to< 95% agreement: 10 points <90% agreement: 15 points		Measures of technical aspects of HIVDR genotyping output
(v) Detection of DRMs	Detection (%) of DRMs in the TG (consensus sequence)	≥95% detected: 0 points 90 to<95% detected: 2 points 85 to<90% detected: 5 points 80 to<85% detected: 10 points <80%: 15 points		
(vi) Reporting of DRMs	DRM(s) reported compared with majority	Five points per DRM not reported		
(vii) Detection of mixtures^a^	Detection (%) of NMs in the TG (consensus sequence)	Not scored		
(viii) Reporting of drug-resistance profile	Agreed interpretation of drug resistance compared with majority when using a single interpretation system, e.g. Stanford Database	Assessed as high, moderate or low level of agreement	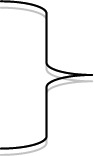	Peer group comparison of drug-resistance profile reported for patient management

H “Hurdle requirement”, participant must return sequence data from at least four of five samples that cluster with same-samples from other participants to be scored on their performance. “No Score” was by definition a poor performance outcome.

a Included to highlight importance of detection of mixtures; not scored as variation in the detection of mixtures(%) was not proportional to detection of DRMs(%).

PR, protease; RT, reverse transcriptase; DRMs, drug-resistance mutations as in IAS-USA list and Stanford Database; TG, target genotype; mixtures, nucleotide mixtures.

At the conclusion of each panel nucleotide sequence alignments, analyses and scores of all participants’ were made available on a password-protected website. Participants were encouraged to review and compare results, and, if necessary, amend and improve their methods. Sequence alignments were provided for participants whose performance was not scored and they were alerted to the reason. Resubmission of results was not permitted.

To provide support and follow-up, annual workshops were conducted for technical and scientific staff directly involved with HIVDR genotyping from participating laboratories, with detailed discussions and expert presentations of laboratory methods. This also facilitated development of a regional network of operators with a range of expertise. When suboptimal performance was identified, laboratories were asked to describe initiatives to address identified problems. In two cases, expert laboratory personnel from within the TAQAS network assisted in setting up HIVDR genotyping protocols or addressed recurring problems.

### Statistical model to predict quality of HIVDR genotyping outcome

To evaluate participants’ HIVDR genotyping outcome, a binary endpoint was defined as detection of ≥95% (high-quality) vs. <95% (low-quality) of consensus DRMs in the TG. Factors potentially associated with *panel complexity* and *laboratory performance* were examined. *Panel complexity* was defined by: (i) number of DRMs in the TG; and (ii) number of DRMs present as mixtures in the TG; (iii) sample subtype (B or non-B); and (iv) sample format (plasma or electropherogram). *Laboratory performance* was defined by: (i) length of nucleotide sequence; (ii) nucleic acid agreement with TG; (iii) detection of DRMs in the TG; (iv) detection of mixtures in the TG; and (v) elements of HIVDR genotyping protocol (i.e. laboratory experience, throughput, time pressure, in-house or outsourced sequencing, nucleotide sequence editing practices and software used). Mixed-effect logistic regression models were used to derive the endpoint using panel complexity and laboratory performance as predictors. All results, including those from incomplete result sets, were included in the models. As laboratories participated in varying panels, and incomplete result sets were included, random-effect models were used to take into account variation both within and between laboratories. The final model included predictors that were significant at 0.10 level (two-sided). Data management and statistical analyses were performed using Stata (StataCorp, STATA 10.1 for Windows, College Station, TX, USA).

## Results

### Participating laboratories

Nine TAQAS panels were delivered to 19 laboratories in the Asia-Pacific, three in Africa and one in the United States (Figure 1). Eight laboratories reported results for all nine panels. Laboratories were recruited over time and on average reported results for six panels (Table 3). Inconsistent participation was due to cessation of HIVDR genotyping services (Lab 11), import restrictions (Lab 13), resource constraints such as reagents, staff or laboratory access shortfall (Labs 14 and 20), or an inability to process electropherograms due to software incompatibility (Labs 3, 7 and 13).

**Table 3:** Panels tested by TAQAS participants over a 4.5-year period

	TAQAS panels								
	
Lab ID	I	II	III	IV^b^	V	VI	VII^b^	VIII	IX
1^a^									
2								DR	
3				NP					
4				DR					
5									
6									
7	NP		DR	DR					DR
8									
9				DR					
10									
11						NP	NP	NP	NP
12	NP			NP	NP		NP		
13	NP	NP		NP					
14	NP	NP	NP			NP		NP	
15	NP	NP	NP						
16	NP	NP	NP						
17	NP	NP	NP	NP	NP				
18	NP	NP	NP						
19	NP	NP	NP	NP	NP				
20	NP	NP	NP	NP	NP	NP		NP	
21	NP	NP	NP	NP	NP			DR	
22	NP	NP	NP	NP	NP	NP			
23^c^	NP	NP	NP	NP	NP	NP	NP	NP	DR

a Stanford University Laboratory

b electropherogram panels

c Lab 23 participated in only one panel; it was included in this presentation to show progressive laboratory enrolment.

NP, laboratory did not participated; DR, data removed from analysis because laboratory returned sequence from <4 of 5 samples or reported outlier sequence for >4 of 5 samples.

## Protocol questionnaire

The Questionnaire, which was completed between the fifth and sixth panels by all but one laboratory (Lab 11 ceased HIVDR testing after Panel VI), demonstrated a wide variability in HIVDR genotyping experience. Laboratories were conducting HIVDR testing for a median of six years (IQR: 5.5 years; range: <1 to 14 years); the median testing throughput was 348 tests per annum (IQR: 625; range: 21 to >4000); and the median per sample turnaround time was 14 days (IQR: 14.5 days; range: 2–30 days). Fourteen laboratories required staff qualifications of a bachelor degree or higher and three required training in molecular biological technique (five did not respond). Time pressure to complete HIVDR testing was perceived by 9 out of 22 laboratories.

The majority of laboratories (18/22) used locally assembled protocols, with wide variations in primers, sequencing probes and input sample volume (data not shown). These aspects of the protocols were not compared. Four laboratories used commercial kits (TruGene^ ®^ one laboratory; ViroSeq^ ®^ three laboratories). The impact on test outcome of participants’ sequencing practices was assessed. Only a few laboratories (6/22) outsourced sequencing. Most laboratories (20/22) used an automatic base calling software and all reported manual checking and editing of automated base calls. In most laboratories (16/22), more than one person was involved in sequence editing. The peak height to call mixed bases was set at 20–30% by 19 laboratories. It was policy in most laboratories (15/22) to review sequence data at sites associated with ARV resistance. Twelve laboratories reported controlling for contamination using software such as Clustal (www.clustal.org) or Mega (www.megasoftware.net).

Of 18 laboratories that used in-house protocols, 15 used the Stanford Database for resistance interpretation and the remaining three used the Stanford Database in conjunction with IAS-USA [24] or ANRS systems [7]. The three laboratories that used Viroseq consulted the Stanford Database in addition to Viroseq guidelines. The laboratory that used TruGene relied solely on the manufacturer's guidelines.

### Laboratory performance

A total of 144 data sets were returned by the 23 participating laboratories; most within the specified turnaround time of five weeks; 10 participants returned results up to five weeks past the turnaround time for one (*n*=9) or two (*n*=1) panels. Late submission of results was recorded and these data sets were included in analyses. One hundred and thirty six data sets (107 plasma and 29 electropherograms) were suitable for assessment. Eight data sets were removed from analyses because they were derived from less than four out of five samples per panel due to sequence amplification failures (two datasets); production of sequence deemed of poor quality (three data sets); or did not satisfy quality control analyses (three data sets) (Table 3). On follow-up, the two laboratories with outlier sequences achieved a non-outlier result by either re-sequencing and changing training protocols; or by preparing new batches of primers and reagents. Revised test outcomes were not re-scored.

**Figure 2:**
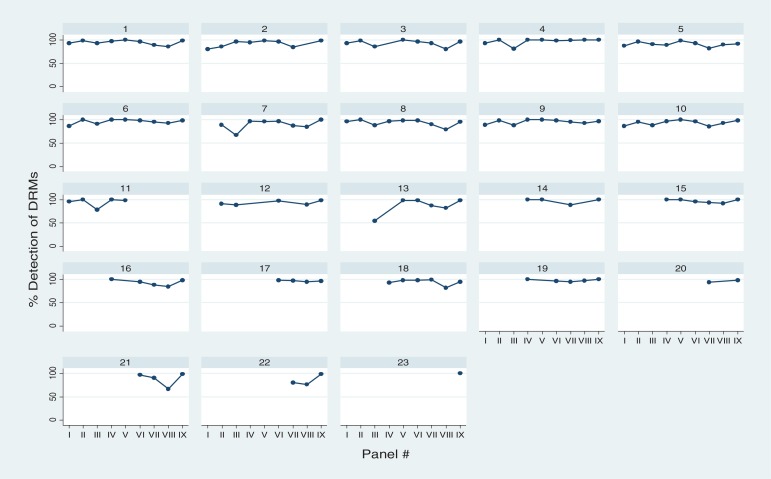
Detection of drug-resistance mutations according to the target genotype (TG) in nine TAQAS panels (one graph per laboratory).

**Figure 3:**
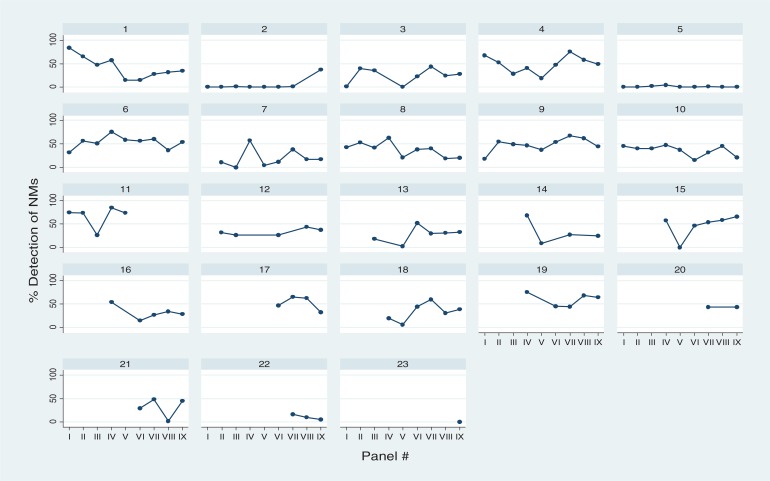
Detection of nucleotide mixtures according to the target genotype (TG) in nine TAQAS panels (one graph per laboratory).

The median detection of DRMs in the TG in the seven plasma panels ranged between 88% and 98% (Table 1). In most reported data sets (102/107), the number of DRMs detected was above the median minus two standard deviations (median–2SD) in each panel. The lowest levels of detection of DRMs were in Panels III (88%), VIII (89%) and I (91%), which had the highest proportion of DRMs presented as mixtures (45%, 30% and 24%, respectively). Although overall detection of DRMs was high throughout the study period, some performance improvement was observed over time. Three laboratories detected <80% of DRMs in early panels but subsequently demonstrated improved performance (Labs 7, 13 and 22: Figure 2). The observed considerable intra- and inter-laboratory variation in the detection of mixtures did not correlate with the percentage of DRMs detected. However, the two laboratories that consistently reported low levels of mixtures or none (Labs 2 and 5; Figure 3) both detected less than the median–2 SD deviations of TG DRMs in two panels, and both had a tendency to underreport the DRMs reported by the majority of participants (data not shown).

The median detection of DRMs in the electropherogram panels was 97% and 90%. Again, detection of mixtures varied (Panel IV: 0–89% with 11 participants; Panel VII: 1–79% with 20 participants). Labs 2 and 5 detected low levels of mixtures in the electropherogram panels (Figure 3). In addition Lab 5's detection of DRMs in Panel 4 was below the mean–2SD. When data from Labs 2 and 5 were removed, the range in the detection of mixtures decreased: Panel IV: 29–89% with 9 participants; Panel VII: 18–79% with 18 participants. TREAT Asia and TAQAS have worked with Labs 2 and 5 addressing software training in detection of mixtures. Lab 2's detection of mixtures increased (from ≤1 to 39% in Panel IX; Figure 3)

### Score as a measurement of performance

Data sets from all participants were quantitatively evaluated using the scoring system (Table 2). High-quality test outcome, defined as detection of ≥95% of the DRMs in the TG, was associated with scoring criteria related to technical aspects of HIVDR genotyping, including adequate phylogenetic clustering, sequence alignment and level of agreement with the TG, and detection of TG DRMs (Table 2, Criteria iii, iv and v; *p*<0.05).

### Predictors of HIVDR genotyping performance

Factors associated with *laboratory performance* that had an impact on HIVDR genotyping outcome included mixture detection (OR: 19.8; *p*<0.001) and level of agreement with the TG (OR: 46.5 for 98-99% agreement, *p*=0.002; and OR: 129.4 for ≥99% agreement, *p*<0.001) (Table 4). Neither length of sequence nor between-laboratory differences in HIVDR genotyping sequencing protocols were associated with performance. Factors associated with *panel complexity* that had an impact on HIVDR genotyping outcome included number of TG DRMs (OR 31.7; *p*<0.001), and number of DRMs present as mixtures (OR: 19.1 for 11-15 DRMs as mixtures, *p*=0.001; and OR: 240 for ≤10 DRMs as mixtures, *p*<0.001). Subtype (B or non-B) and sample format (plasma or electropherogram) were not associated with genotyping performance.

**Table 4:** Predictors of high-quality HIV drug-resistance genotyping outcome

Covariate	Odds ratio (95% CI)	*p*
Laboratory performance^a^		
Mixtures detected (%)		
≤50^b^	1.00	
>50	19.78 (4.16, 94.09)	<0.001
Agreement with nucleotide sequence		
<98%^b^	1.00	
98 to<99%	46.54 (3.87, 559.51)	0.002
≥99%	129.41 (10.32, 1622.32)	<0.001
Panel complexity		
Number of DRMs as mutants		
≤40^b^	1.00	
>40	31.66 (6.92, 144.81)	<0.001
Number of DRMs as mixtures		
>15^b^	1.00	
11–15	19.10 (3.43, 106.39)	0.001
≤10	239.95 (27.89, 2064.56)	<0.001

a Compared with target genotype (TG)

b reference category.

## Discussion

We report extended results from TAQAS, an EQA programme for HIVDR genotyping by a group of laboratories in Asia-Pacific and Africa [20]. Twenty-two laboratories (not including the certified US laboratory) from 13 countries demonstrated proficient HIVDR genotyping of 45 HIV-1 multi-subtype samples in nine panels, as evidenced by a low level of amplification failure; minimal sample cross-contamination; high levels of DRMs detection and sequence homology to consensus sequences; and compliance with a test turnaround time indicating provision of results in a clinically relevant timeframe. Intra-and inter-laboratory variation in detection of mixtures was observed, and in some laboratories was associated with sub-optimal detection and reporting of DRMs. HIVDR genotyping execution was associated with panel complexity factors including numbers of DRMs and DRMs occurring as mixtures, and with laboratory performance factors including detection of mixtures and agreement with TG, but not with differences in laboratories’ use of commercial vs. in-house tests or sequencing protocols. A new scoring system showed that quality of test outcome was related to technical proficiency in HIVDR genotyping. In contrast to other EQA programmes [8, 22, 25–28], TAQAS participants were supported to address testing deficiencies, and their performance improved in subsequent panels. The programme's feedback in response to suboptimal performance and its educationally oriented approach may have contributed to the high quality of the testing outcomes.

Similar to other EQA programmes, TAQAS participants varied in testing experience and access to technical resources [25, 29]. In contrast to other studies where laboratories predominantly used commercial kits and consensus protocols and no difference in test outcome was found, the majority of TAQAS participants used a variety of in-house technologies, as well as several sequence editing software programmes [5, 25, 30, 31]. However, most participants had similar sequence editing practices and used the Stanford Database for interpretation. Based on these data, low-cost in-house assembled assays can successfully be used for HIVDR testing with the support of EQA programmes.

EQA programmes should aim to assess the HIVDR laboratory testing process using clinically relevant sample types. The predominant use of plasma samples in TAQAS enabled assessment of detection of viral mixtures, important in DRM detection [6, 8, 20, 32], and useful in the assessment of inter-laboratory testing variation [33]. The use of a sample type with inherent variability like plasma, in contrast to clones or plasmids, mandated the use of a TG rather than a sequence derived by a reference laboratory [10, 25, 30]. While inter-laboratory comparison is complicated by inclusion of samples with mixtures in EQA panels, the laboratory's ability to detect mixtures is an important measure of the quality of the genotyping output and every effort should be made to monitor and support this in an EQA programme [5].

Subtle differences in EQA programmes that reported higher levels of DRM detection than reported by TAQAS participants are noted [25, 27, 29–31]. Some programmes report on the distribution of a single panel or low-sample-number panels, and/or use virus derived from viral culture supernatant, cloned or extracted material as the sample format. Such programmes could expect higher detection levels of DRMs compared to those seen with clinical samples, due to differences in sample variability. Indeed, comparable detection of DRMs to that seen in TAQAS was reported in a four-plasma-sample distribution to 20 laboratories [28]. Similarly, panels incorporating non-subtype B samples, as in TAQAS, can affect sequence variability and TG concordance, due to inter-subtype genetic differences. Such samples should be included in EQA panels, particularly for laboratories in settings where non-B subtypes predominate.

Panel complexity, detection of mixtures and level of sequence agreement were found to predict HIVDR genotyping quality, confirming previous reports [5, 20, 25, 28, 30, 34, 35].

The electropherogram samples used in two TAQAS panels showed that variation in detection of mixtures can occur because of inter-laboratory differences post-sequence production, rather than genotyping extraction, amplification and sequencing protocols. Sequence editing has been suggested to contribute to disparity in HIVDR genotyping outcomes particularly with respect to the mixtures detection [36]. However, while there was variation in sequence editing software used, most participants adhered to common, high standards to edit raw sequence data. This may account for the lack of association between the quality of the test outcome and sequence editing practices demonstrated by this group. Sequences with a high number of mixtures should be incorporated into EQA panels, and laboratories should be encouraged to develop proficiency in mixture calling, which is directly related to identification of DRM.

Objective assessment of EQA performance outcomes enables intra- and inter-laboratory and between-panel comparison, and potentially comparison between EQA programmes [5, 25, 31, 37]. The TAQAS scoring system described here extends systems previously reported, by adding a measure of clinical utility; a hurdle requirement of the production of good quality, sample-specific sequence thereby emphasizing the importance of quality control measures as performance indicators; detection and reporting of all consensus DRMs and DRMs presented as mixtures; and the interpretation of ARV resistance as per the peer group majority [5, 25, 26, 31, 37]. Scores on technical aspects of testing were associated with the quality of test outcome. Scoring multiple test components improved the value of the EQA exercise for participants by flagging possible causes of suboptimal performance. Objective measurement of test outcomes enables fair, on-going assessment of participants’ EQA outcomes that may be required for auditing, by funding bodies and for participation in multi-centre clinical trials.

Some previously reported limitations of TAQAS [20] have been addressed here. Information about participants’ testing procedures was documented, the number of participants more than doubled and criteria against which HIVDR genotyping outcomes were assessed were defined. Existing limitations include lack of ability to compare genotyping technologies among participants, as most used in-house assays [25]. These are realistic circumstances in cost-constrained settings. The inclusion of non-B and recombinant viruses relevant to Asia has increased, though not sufficiently to assess the impact of different subtypes on test outcomes [30]. The importance of including such samples is acknowledged [5] although other EQA providers have provided predominantly subtype B samples [30, 31, 35].

TAQAS was established to build capacity for and assure quality of HIVDR genotyping to support clinical care and research in Asia and Africa. Participation by 22 laboratories over an extended timeframe confirmed their HIVDR testing proficiency. Several reasons are proffered as to the high-quality test outcomes of this complicated test: diligent and conscientious attitude of participants, provision of complex clinical samples, comprehensive and comparative analysis of results, follow-up initiatives after suboptimal performance, and support and information dissemination by TREAT Asia and NRL. TAQAS facilitated successful application of a quantitative measure of laboratory performance of HIVDR genotyping and identification of predictors of test quality. Both tools can improve utility of future EQA programmes. The importance of continuous EQA participation to maintain and improve HIVDR genotyping outcome has been validated [8, 25, 30]. Recent reports provide novel methods to standardize the interpretation of electropherograms for HIVDR testing [38, 39]. Though promising, it will take time until such methods are incorporated into laboratories’ protocols. As methods are developed and modified, their incorporation into quality assessment programmes will be essential. As demonstrated by TAQAS, quality assessment programmes not only assess proficiency but can also be harnessed to establish, expand and improve testing, and be used as a vehicle for educational initiatives and the creation of collaborative and educational testing laboratory networks.
